# Impact of *in vitro* HIV infection on human thymic regulatory T cell differentiation

**DOI:** 10.3389/fmicb.2023.1217801

**Published:** 2023-07-20

**Authors:** Sharada Swaminathan, Tatiana Scorza, Alexis Yero, Omar Farnos, Stephanie C. Burke Schinkel, Jonathan B. Angel, Mohammad-Ali Jenabian

**Affiliations:** ^1^Department of Biological Sciences and CERMO-FC Research Centre, Université du Québec à Montréal (UQAM), Montreal, QC, Canada; ^2^Division of Infectious Diseases, Ottawa Hospital-General Campus, Ottawa, ON, Canada; ^3^Ottawa Hospital Research Institute, Ottawa, ON, Canada

**Keywords:** HIV, thymus, regulatory T cells, Treg, thymocytes, FoxP3, TGF-β

## Abstract

**Background:**

The differentiation and function of immunosuppressive regulatory T cells (Tregs) is dictated by the master transcription factor FoxP3. During HIV infection, there is an increase in Treg frequencies in the peripheral blood and lymphoid tissues. This accentuates immune dysfunction and disease progression. Expression of FoxP3 by thymic Tregs (tTregs) is partially controlled by TGF-β. This cytokine also contributes to Treg development in the peripheral blood and lymphoid tissues. Although TGF-β mediates lymphoid tissue fibrosis and peripheral Treg differentiation in HIV-infected individuals, its role in the induction and maintenance of Tregs within the thymus during HIV infection remains unclear.

**Methods:**

Thymocytes were isolated from fresh human thymic tissues obtained from pediatric patients undergoing cardiac surgery. Infection by both R5- and X4-tropic HIV-1 strains and TGF-β treatment of human thymocytes was performed in an *in vitro* co-culture model with OP9-DL1 cells expressing Notch ligand delta-like 1 without T cell receptor (TCR) activation.

**Results:**

Despite high expression of CCR5 and CXCR4 by tTregs, FoxP3 +  CD3^high^CD8- thymocytes were much less prone to *in vitro* infection with R5- and X4-tropic HIV strains compared to FoxP3-CD3^high^CD8- thymocytes. As expected, CD3^high^CD4+ thymocytes, when treated with TGF-β1, upregulated CD127 and this treatment resulted in increased FoxP3 expression and Treg differentiation, but did not affect the rate of HIV infection. FoxP3 expression and Treg frequencies remained unchanged following *in vitro* HIV infection alone or in combination with TGF-β1.

**Conclusion:**

FoxP3 expression and tTreg differentiation is not affected by *in vitro* HIV infection alone or the combination of *in vitro* HIV infection and TGF-β treatment.

## Background

Immunosuppressive regulatory T cells (Tregs) are responsible for the induction of immune tolerance to prevent autoimmunity and for the maintenance of immune homeostasis (Sakaguchi et al., 1995; Seddon, 2000; Kim et al., 2007; Sakaguchi et al., 2013). The expression of several genes governing the differentiation of Tregs, the maintenance of the phenotype of these cells, as well as their suppressive functions (Fontenot et al., 2005; Sakaguchi et al., 2013), is dictated by the protein FoxP3. This transcription factor is therefore referred to as the “master regulator” of Tregs. Mature human Tregs are described phenotypically as CD3^+^CD4^+^CD25^high^CD127^low^FoxP3^high^ (Jenabian et al., 2012; Chevalier and Weiss, 2013; Sakaguchi et al., 2013). Tregs may have two developmental origins: (1) natural or thymic Tregs (tTregs), which originate within the thymus as a T-cell lineage and (2) induced Tregs (iTregs), which are derived from peripheral naive CD4+ T-cells that start expressing FoxP3 under inflammatory conditions and/or *in vitro,* upon stimulation with Transforming Growth Factor Beta (TGF-β) (Chen et al., 2003; Curotto de Lafaille et al., 2004; Curotto de Lafaille and Lafaille, 2009; Sakaguchi et al., 2013). Within the thymus, tTreg development occurs in two main steps: survival of thymocytes bearing TCR with relatively high affinity to self-antigens and cytokine-dependent induction and maintenance of FoxP3 expression (Lio and Hsieh, 2008). TGF-β is a pleiotropic cytokine, involved in many biological proceeses such as differentiation of cells, their proliferation, embryonic development, carcinogenesis, fibrosis, tissue repair and healing, and angiogenesis (Blobe et al., 2000). In mammals, there are three different isoforms of TGF-β (TGF-β1, TGF-β2, and TGF-β3), each encoded by a different gene (Govinden and Bhoola, 2003). The TGF-β1 isoform is implicated in the regulatory aspects of the immune response (Li et al., 2006). TGF-β is also known to regulate FoxP3 expression during tTreg development and inflammation in other tissues, notably in gut lymphoid tissues (Chen et al., 2003; Li and Flavell, 2008; Konkel and Chen, 2011; Zeng et al., 2011; Chen and Konkel, 2015; Jurberg et al., 2015).

It is well documented that the proportion of Tregs in blood and secondary lymphoid organs is increased during HIV/SIV infections (Andersson et al., 2005; Bi et al., 2009; Zeng et al., 2011; Jenabian et al., 2012; Moreno-Fernandez et al., 2012; Chevalier and Weiss, 2013; Kleinman et al., 2018; Yero et al., 2019). Importantly, in HIV/SIV infections, Treg accumulation in lymphoid tissues has been directly correlated with the severity of the disease and immunodeficiency (Nikolova et al., 2011; Zeng et al., 2011; Jenabian et al., 2012; Moreno-Fernandez et al., 2012; Chevalier and Weiss, 2013; Kleinman et al., 2018; Yero et al., 2023). This phenomenon is most obvious in the gut associated lymphoid tissue (GALT), where the increase in proportion of Tregs results in an imbalance between Tregs and T helper 17 (Th17) cells, which results in impaired mucosal immune responses, which consequently leads to microbial translocation and exacerbated systemic activation of the immune system (Favre et al., 2009; Kim et al., 2013; Maartens et al., 2014; Yero et al., 2023). Indeed, even under effective antiretroviral therapy (ART), immunosuppressive Tregs can inhibit both the HIV-specific immune responses and the proliferation of effector CD4 and CD8 T-cells (Aandahl et al., 2004; Nilsson et al., 2006; Favre et al., 2009; Zeng et al., 2011; Jenabian et al., 2012; Chevalier and Weiss, 2013; Jenabian et al., 2013; Kim et al., 2013; Maartens et al., 2014; Kleinman et al., 2018; Yero et al., 2019). Our team reported a decrease in the proportions of Tregs in the peripheral blood following early initiation of ART in acute HIV infection compared to healthy controls (Jenabian et al., 2015; Yero et al., 2021). Using SIV-infected rhesus macaques, we demonstrated that even early ART initiation cannot prevent the increase in Treg frequencies or migration of Tregs into mesenteric lymph nodes, thereby contributing to immune dysfuction in the gut, and probably also leading to fibrosis and reduced functioning of these lymph nodes (Yero et al., 2019). This negative role of Tregs during HIV infection is mostly via the production of TGF-β. Indeed, Tregs producing TGF-β are implicated in collagen deposition and gut lymphoid tissue fibrosis and immune dysfunction, which in turn, causes generalized inflammation and HIV/SIV disease progression (Estes et al., 2007; Zeng et al., 2011; Jenabian et al., 2012; Moreno-Fernandez et al., 2012; Chevalier and Weiss, 2013; Yero et al., 2019, 2023). Therefore, a rational approach to predictably inhibit Treg development and function could be an important therapeutic tool in the context of HIV infection. However, this requires a better understanding of the mechanisms of differentiation and activation of tTregs versus iTregs during HIV infection.

Thymic involution has been reported in chronically HIV-infected individuals and in children with AIDS (Grody et al., 1985; Joshi et al., 1986; Haynes et al., 1999). Indeed, thymocytes and thymic dendritic cells are susceptible to HIV infection and, consequently, their immunological and biological functions are altered by HIV infection (Evans et al., 2011; Young and Angel, 2011; Nunes-Cabaço et al., 2015). Importantly, SIV infection of rhesus macaques showed that lymphocytes and macrophages within the thymus are infected early (within 2 weeks) by SIV (Baskin et al., 1991). However, the role of the thymus and the mechanisms of increased Treg frequencies during HIV infection have not yet been well described. It is plausible that besides inducing peripheral Treg generation, TGF-β might play a part in the induction and maintenance of Tregs in the thymus during HIV infection. Hence, we hypothesized that TGF-β induces FoxP3 expression in HIV-infected thymocytes and thus contributes to higher thymic Treg frequencies during HIV infection. Herein, we assessed the impact of *in vitro* HIV infection of human thymocytes on the differentiation of tTregs and the influence of TGF-β1 in a co-culture model with OP9-DL1 cells expressing Notch ligand delta-like 1.

## Materials and methods

### Human thymus tissues and isolation of thymocytes

Portions of fresh human thymic tissue (usually between a quarter to half of the thymus with an average size of 2–4 cm^3^) were obtained from both male and female children (less than 1 year old) undergoing cardiac surgery at the Children’s Hospital of Eastern Ontario (CHEO), Ottawa, ON, Canada. Thymic tissue was cut into small sections, placed on 70 μm cell strainers (ThermoFisher Scientific, Canada), and dipped in sterile RPMI (Wisent, Canada) with 10% fetal bovine serum (FBS) (Wisent) and 1% penicillin–streptomycin (Wisent). Tissue pieces were then mechanically disrupted, and cell suspensions were collected and washed with RPMI. The cell pellet obtained was re-suspended and thymocytes were purified by Ficoll centrifugation (Wisent). Purified thymocytes (between 1–2 billion cells) were either cryo-preserved or used immediately for experiments.

### Ethical considerations

The study was conducted in compliance with the principles included in the Declaration of Helsinki. Approval for this study was obtained from the Research Ethics Board of CHEO Research Institute and Université du Québec à Montréal (UQAM, #216_e_453). Written informed consent was obtained from the parents of all children from whom thymic tissues were obtained.

### HIV-1 viral stocks

Plasmids of CCR5-tropic (110NB) and CXCR4-tropic (NL4.3) HIV-1 viral strains were obtained from the NIH. Stocks of viral strains were generated in the laboratory by transfecting HEK293T cells with the plasmids. Viral concentration in the supernatants was measured by p24 ELISA as previously described (Gosselin et al., 2010), and was then used for thymocyte infection.

### Flow cytometry

**Figure 1:**
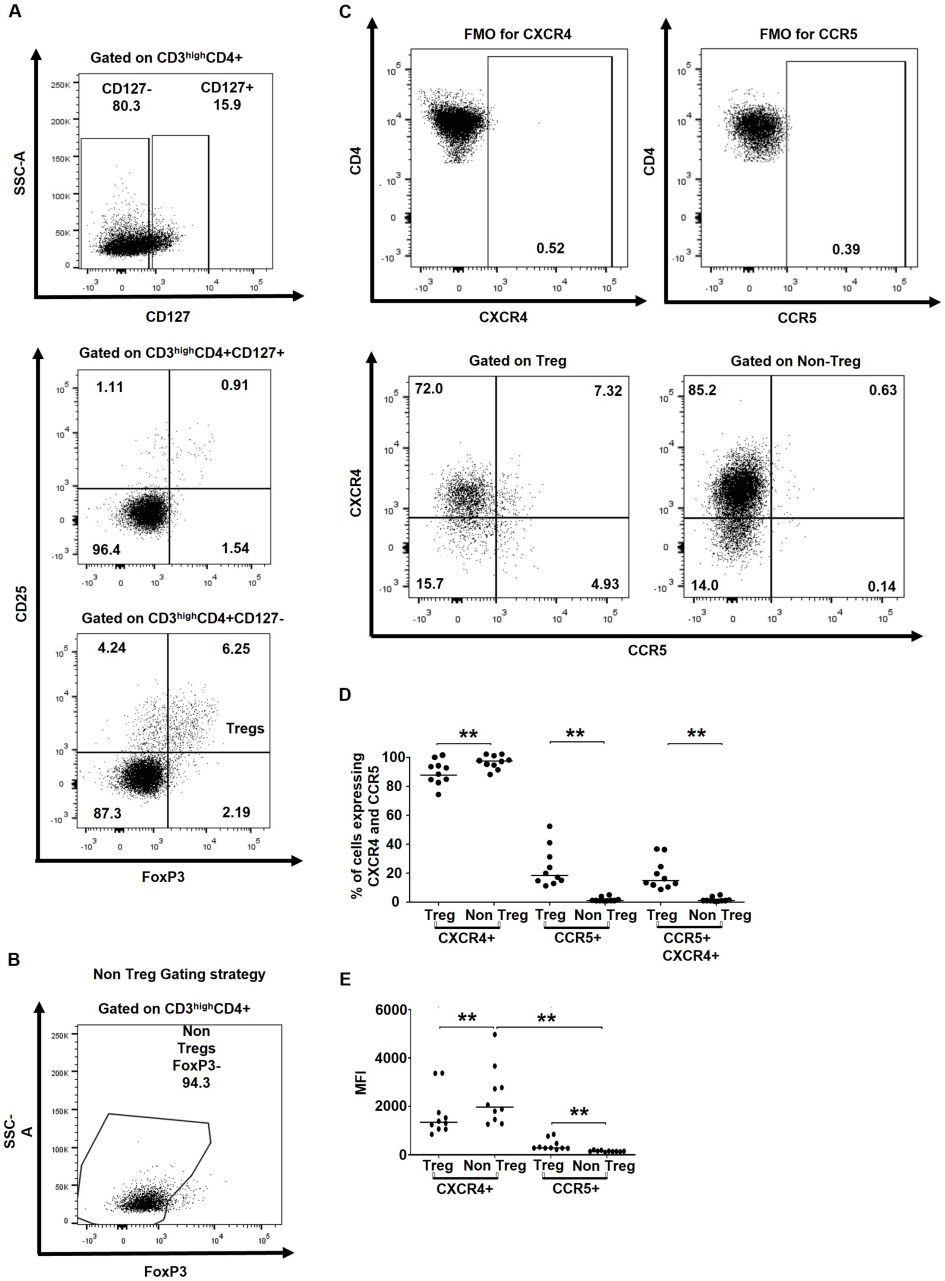
tTregs express higher levels of HIV co-receptors CXCR4 and CCR5. **(A)** Gating strategy used to characterize tTregs (CD3^high^CD4 + CD8-CD127-CD25 + FoxP3+) within CD3^high^ thymocytes. **(B)** Gating strategy used to characterize non-Tregs (CD3^high^CD4 + CD8-FoxP3-) within CD3^high^ thymocytes. **(C)** Gating strategy for determining CXCR4 and CCR5 expression on Tregs and non-Tregs. **(D)** The *ex vivo* frequencies of thymocytes expressing CXCR4 and/or CCR5. **(E)** The *ex vivo* MFI of the CXCR4 and CCR5 expression. Data from *n* = 10 thymi are presented. Only significant Wilcoxon statistical *p*-values are included within the figure. ** *p* ≤ 0.01. n.s, non significant. *Nota bene*: The gating strategy for CD4+ cells excluded CD4 + CD8+ thymocytes.

For both *ex vivo* and *in vitro* experimental settings, 1 × 10^6^ thymocytes were resuspended in 250 μL of FACS-staining buffer (1X PBS + 2% FBS). Cells were then incubated with antibody cocktails for 45–60 min for extracellular staining and washed with 3 mL of 1X PBS. The BD transcription buffer kit (BD Pharmigen, New Jersey, United States) was used to permeabilize the cells prior to intracellular staining. The following antibodies from BD Biosciences were used for extracellular staining: CD3-Alexafluor700 (Clone: UCHT1), CD4-FITC (Clone: RPA-T4), CD4-BV650 (Clone: L200), CD8-APC-H7 (Clone: SK1), CD25-BV786, CD25-PE (Clone: M-A251), CD127-PE-Cy7 (Clone: HIL-7R-M21), CD184 (CXCR4)-BV711 (Clone: 12G5), CD195 (CCR5)-Alexafluor488 (2D7/CCR5). FoxP3-PECF594 (Clone: 236A/E7) from BD Biosciences and p24-PE (Clone: KC57-RD1) from Beckman Coulter were used for intracellular staining. The LIVE/DEAD™ Fixable Green Dead Cell Stain Kit, for 405 nm excitation from ThermoFisher, was also used. Flow cytometry acquisition and analysis have been performed using BD LSR Fortessa-X20 and FlowJo V10.2 software (Ashland, Oregon, United States) respectively. To determine the position of the gates, subpopulations of cells within each sample that do not express a chosen marker were used as internal controls. For instance, the CD3^neg^ subset does not express FoxP3 and hence was used as a negative internal control to determine the position of the gate. FMOs were used to set the gates for CXCR4 and CCR5 (Figure 1).

### Preparation of primary human thymocytes for *in vitro* assays

*In vitro* assays were performed using either freshly isolated or thawed thymocytes. Thawed thymocytes were treated with DNase I (STEMCELL, Vancouver, Canada) for 15 min to prevent cell clumping. Dead cells were separated from live cells by staining with LIVE/DEAD™ Fixable Green Dead Cell Stain Kit, for 488 nm excitation (ThermoFisher) for 30 min and sorting with BD FACSJazz™ cell sorter. Live cells were collected in FBS and were used for cell culture.

### Co-culture model of human thymocytes and OP9-DL1 cell line

In order to avoid the impact of TCR stimulation on the expression of FOXP3, all *in vitro* stimulation of human thymocytes and HIV infection experiments were carried out using a co-culture model with the mouse stromal cell line OP9-DL1 – which expresses the ligand for Notch, delta-like-1 (DL-1) as we previously reported (Young and Angel, 2012). Notch signaling is essential for T cell differentiation and lineage commitment in the thymus. A previous study has reported that *in vitro* differentiation of T-cells is achieved by co-culturing hematopoietic or embryonic stem cells with OP9-DL1 (Holmes and Zúñiga-Pflücker, 2009). This cell line was generously provided by Dr. Zúñiga-Pflücker, University of Toronto. The co-culture has been established with a ratio of thymocyte: OP9- DL1 of 25:1 (Young and Angel, 2012). OP9-DL1 cells were maintained in culture in AMEM (Wisent) containing 20% FBS and 1% penicillin–streptomycin and passaged every 2–3 days using trypsin–EDTA (Wisent). In some experiments on thawed thymocytes, to achieve optimal cell viability (>95%) that is comparable to fresh specimen, after treatment with DNase I for 15 min, we eliminated the dead cells using a Dead Cell Removal (Annexin V) Kit (STEMCELL, Vancouver, Canada). Of note, high rates of cell death are inherent to thymocytes development via negative and positive selection (Hernandez et al., 2010), therefore it is expected to observe elevated mortality in both fresh and frozen/thawed samples.

### *In vitro* HIV infection and TGF-β stimulation of thymocytes

Our team has previously reported that OP9- DL1 co-culture is an optimal model for *in vitro* stimulation and HIV infection (Young and Angel, 2011; Young and Angel, 2012). One million of freshly isolated or thawed thymocytes were treated with polybrene (Sigma Aldrich, Canada) at 3 μg per 10^6^ cells for 1 h, at 37°C to increase the infection rate. Media with polybrene was then removed and laboratory-constructed strains of R5 or X4 HIV-1 viral stocks were added to the cells at a concentration of 50 ng p24/mL per 1 × 10^6^ cells. The plates were centrifuged at 1200 r.c.f for 3 h at 25°C to facilitate infection (spinoculation). After 3 h, cells were washed twice with media, and cultured in the presence or absence of recombinant human TGF-β1 (Abcam, Cambridge, United Kingdom) at 10 ng/mL and co-cultured with OP9- DL1 in RPMI with 10% FBS and 1% penicillin–streptomycin for 2 days. In some experiments, treatment with the TGF-β1 inhibitor Pirfenidone (PFD, 2.6 mM, Selleck chemicals, Houston, TX) for 1 h prior to the addition of TGF-β1 was performed. After 2 days of culture post-infection, cells were stained for flow cytometry or preserved in Trizol for mRNA extraction and qPCR. The time-point of 2 days of co-culture has been selected based on cell viability and HIV infection rate of thymocytes assessed by intra-cellular p24 staining as shown in Supplementary Figure 1.

### Quantification of TGF-β mRNA by qPCR

RNA from 10^6^ thymocytes was extracted by the Trizol-chloroform method (TRIzol™ Reagent, Invitrogen, OR, United States). RNA concentration was measured using the NanoDrop (ThermoFisher Scientific, Canada). RNA concentrations were normalized in all samples before cDNA synthesis. To protect RNA from degradation by RNAses, RNaseOUT™ (Invitrogen, OR, United States) recombinant ribonuclease inhibitor was used. qPCR samples were prepared using LightCycler^®^ 480 SYBR Green I Master kit (Roche, Canada) in white, 96 well, LightCycler Plates, (Progene PCR plates, UltiDent, Canada) and covered with plate seals (Invitrogen). The following primers specific to human cDNA were used: TGF-β1 F: TACCT GAACCCGTGTTGCTC, R: AGTGAACCCGTTGATGTCCA, TGF-β2 F: GTGCTCTGTGGGTACCTTGAT, R: ATCCCAGGTTCC TGTCTTTATGG, TGF-β3 F: CCGAGTGGCTGTCCTTTGAT, R: TCCTCATTGTCCACGCCT, β-Actin: F: CCCTGGAGAAGAGC TACGAG, R: CGTACAGGTCTTTGCGGATG. Expression levels of the gene of interest were determined relative to the expression level of the housekeeping gene β-actin. The plates were run in LightCycler^®^ 480 instrument (Roche, Switzerland) with the following program: 95°C for 5 min, then 40 amplification cycles of 15 s at 95°C, 20 s at 59°C (62°C for TGF- β2) and 20 s at 72°C; one cycle of 5 s at 95°C, and 30 s at 65°C; and 10 s at 40°C.

### Statistical analysis

Statistical analyses were performed using GraphPad version 6.01 (California, United States). Wilcoxon matched-pairs signed-rank test was used to compare paired study variables and median values are presented throughout the manuscript. *p* values are shown only in cases where the differences were significant (*p* < 0.05).

## Results

### Differential expression of HIV co-receptors CXCR4 and CCR5 by tTregs compared to non-Treg thymocytes

To assess the potential susceptibility of tTregs versus non-Treg CD4+ thymocytes, we first evaluated their *ex vivo* expression of HIV co-receptors CXCR4 and CCR5 by flow cytometry. tTregs were identified as CD3^high^CD4 + CD127^low^CD25 + FoxP3+, whereas non-Treg thymocytes were defined as CD3^high^CD4 + FoxP3- cells (Figures 1A–C). In line with previous studies (Schmitt et al., 2006; Nunes-Cabaço et al., 2015), the majority of tTreg and CD4 + FoxP3- non-Tregs expressed CXCR4, while the percentage of CCR5+ and CCR5 + CXCR4+ cells were significantly higher in Tregs compared to non-Tregs (Figure 1D). Similarly, MFI values for CXCR4 were higher in non-Tregs, whereas MFI values for CCR5 were higher in tTregs (Figure 1E).

### FoxP3+ thymocytes are less susceptible to *in vitro* HIV infection than FoxP3- thymocytes

**Figure 2:**
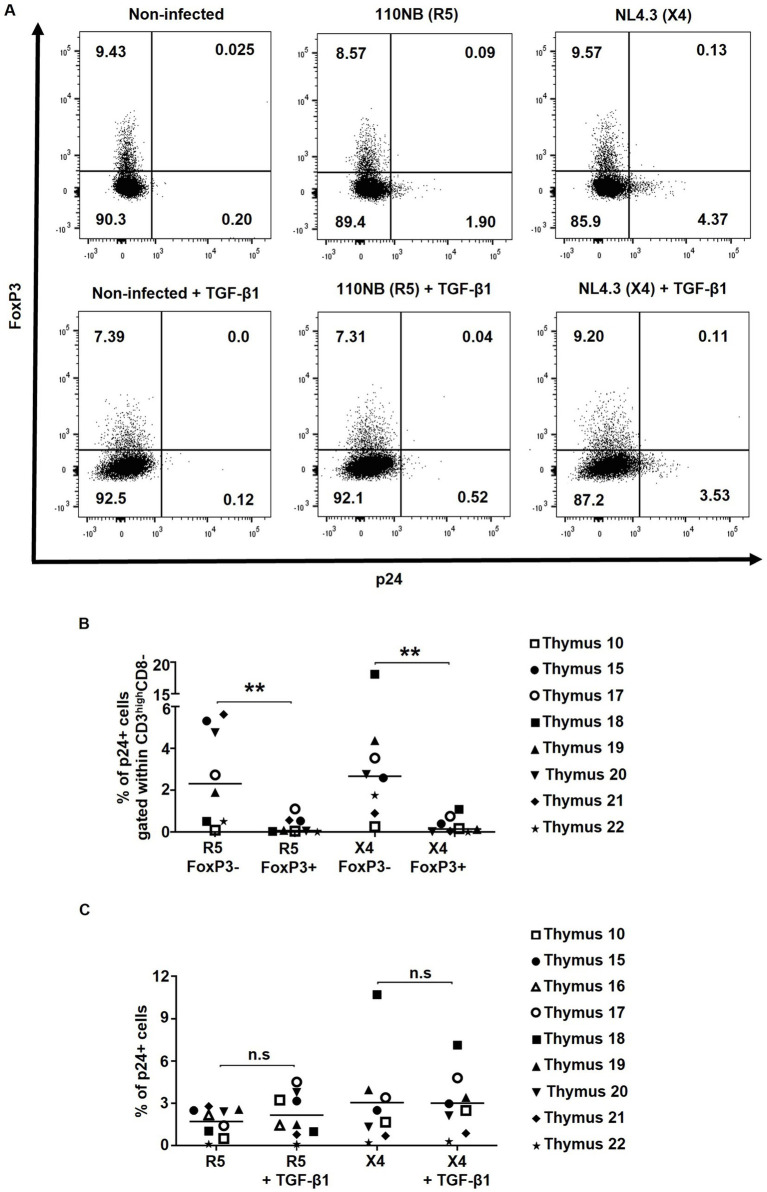
FoxP3+ thymocytes are less susceptible to *in vitro* HIV infection compared to FoxP3- thymocytes. Human thymocytes were infected with R5-tropic (110NB) and X4-tropic (NL4.3) HIV-1 viral strains for 3 h. **(A)** The infection rates achieved with R5 and X4 viral strains were determined by the expression of HIV capsid protein p24 in FoxP3+ and FoxP3- thymocytes gated on alive CD3^high^CD8- cells. **(B)** Frequencies of p24+ cells within CD3^high^CD8-FoxP3- and CD3^high^CD8-FoxP3+ cells are shown. **(C)** The frequencies of p24+ within CD3^high^CD8- in non-treated or TGF-β-treated samples are shown. Data from *n* = 9 thymi are presented. * *p* ≤ 0.05, ** *p* ≤ 0.01. n.s, non significant.

To measure the infection rate of thymocytes with R5- and X4-tropic HIV strains, and considering the known downregulation of CD4 upon HIV infection (Kwon et al., 2020), the percentages of p24+ cells within FoxP3+ CD3^high^CD8- and FoxP3-CD3^high^CD8- thymocytes were assessed for co-expression of FoxP3 and p24 (Figure 2A) at day 2 post-infection. Longer incubation periods were associated with increased mortality and very low infection rate and thus were not used for this *in vitro* assay (Supplementary Figure 1) (Kwon et al., 2020). Upon thymocyte infection with the R5- and X4-tropic HIV strains, a very low infection rate was observed in CD3^high^CD8- thymocytes co-expressing FoxP3 and p24 (Figures 2A,B), suggesting that despite expressing high levels of the HIV co-receptors CXCR4 and CCR5, FoxP3+ thymocytes are less susceptible to *in vitro* HIV infection.

### Effect of TGF-β1 on *in vitro* HIV infection of thymocytes and tTreg differentiation

To assess the impact of TGF-β1 treatment on HIV infection, p24 expression was compared in TGF-β1-treated HIV-infected thymocytes versus non-treated controls. Herein, no significant differences in the frequencies of p24 + CD3^high^CD8- thymocytes were found following TGF-β1 treatment (Figures 2A,C), suggesting that TGF-β1 does not modulate HIV infection of thymocytes *in vitro.*

**Figure 3:**
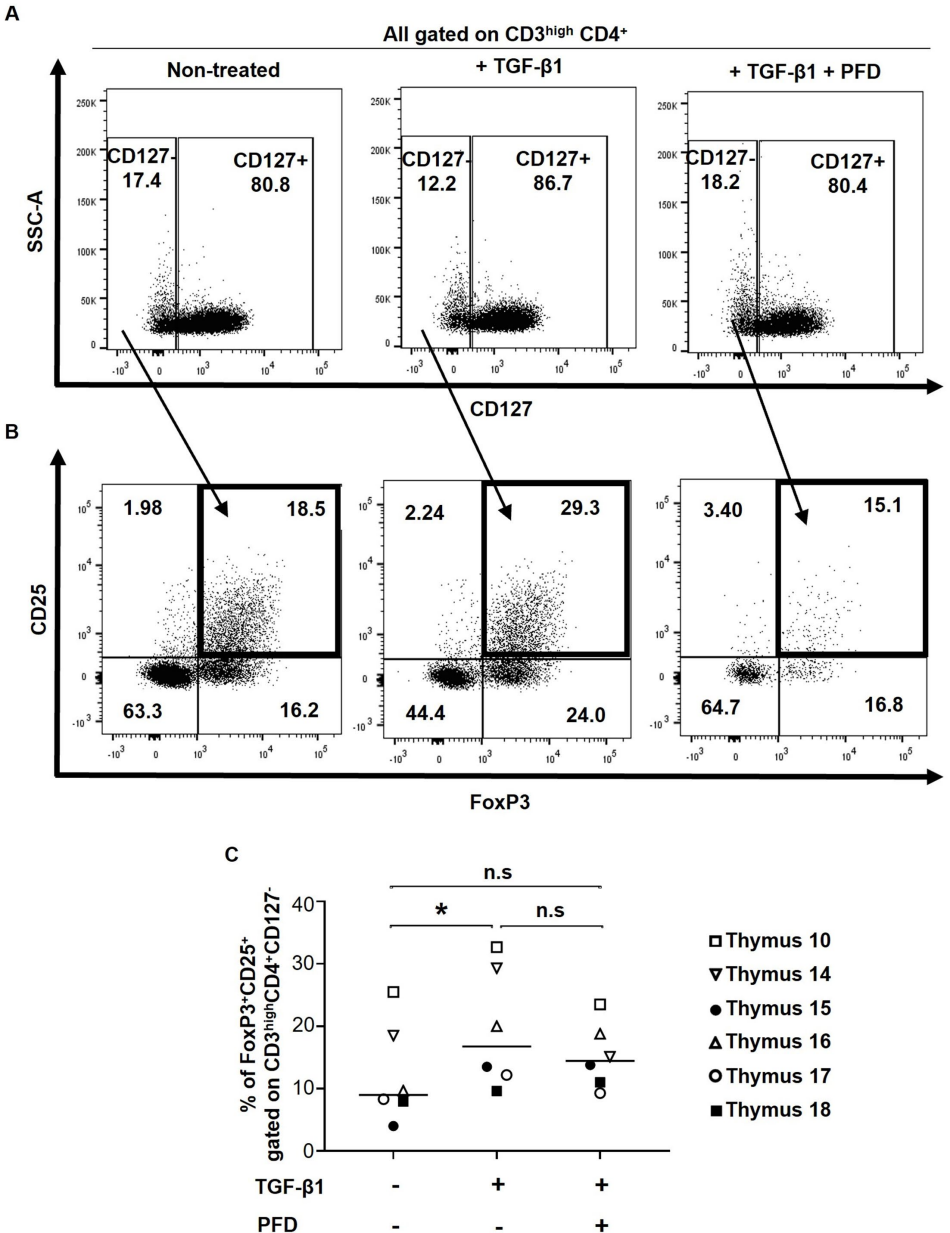
Impact of TGF-β1 treatment on thymic Treg frequencies. Human thymocytes were conditioned with TGF-β in the presence or absence of Pirfenidone (PFD). **(A)** Effect of TGF-β1 and PFD treatment on CD127 expression. **(B)** Thymic Tregs were characterized as CD3^high^CD4 + CD127-CD25 + FoxP3+ cells. The gating strategy for CD4+ cells excluded CD4+ CD8+ thymocytes. **(C)** Frequencies of Tregs following TGF-β1 and PFD treatment. Data from *n* = 6 thymi are presented. * *p* ≤ 0.05. n.s, non significant.

To investigate the potential impact of TGF-β1 in tTreg differentiation, 1×10^6^ human thymocytes were treated with TGF-β1, and in some conditions, the TGF-β1 inhibitor PFD was also added to the culture 1 h prior to TGF-β1 treatment. A slight, although not significant decrease in cell viability was observed following the treatment with TGF-β1 and PFD (data not shown). Considering that TGF-β is known to increase CD127 expression (Ouyang et al., 2013), expression of this marker was used as an internal control for TGF-β1 functionality. As previously reported (Ouyang et al., 2013), treatment with TGF-β1 resulted in higher expression of CD127 in CD4+ thymocytes and PFD reversed this effect (Figure 3A). Importantly, treatment with TGF-β1 significantly increased the frequencies of tTregs (CD3^high^CD4^+^CD127^−^CD25^+^FoxP3^+^) in all samples, an effect that was blocked by PFD (Figures 3B,C). Altogether, our results validate and highlight the role of TGF-β1 in FoxP3 expression and Treg differentiation.

### *In vitro* HIV infection does not affect FoxP3 expression in thymocytes

**Figure 4:**
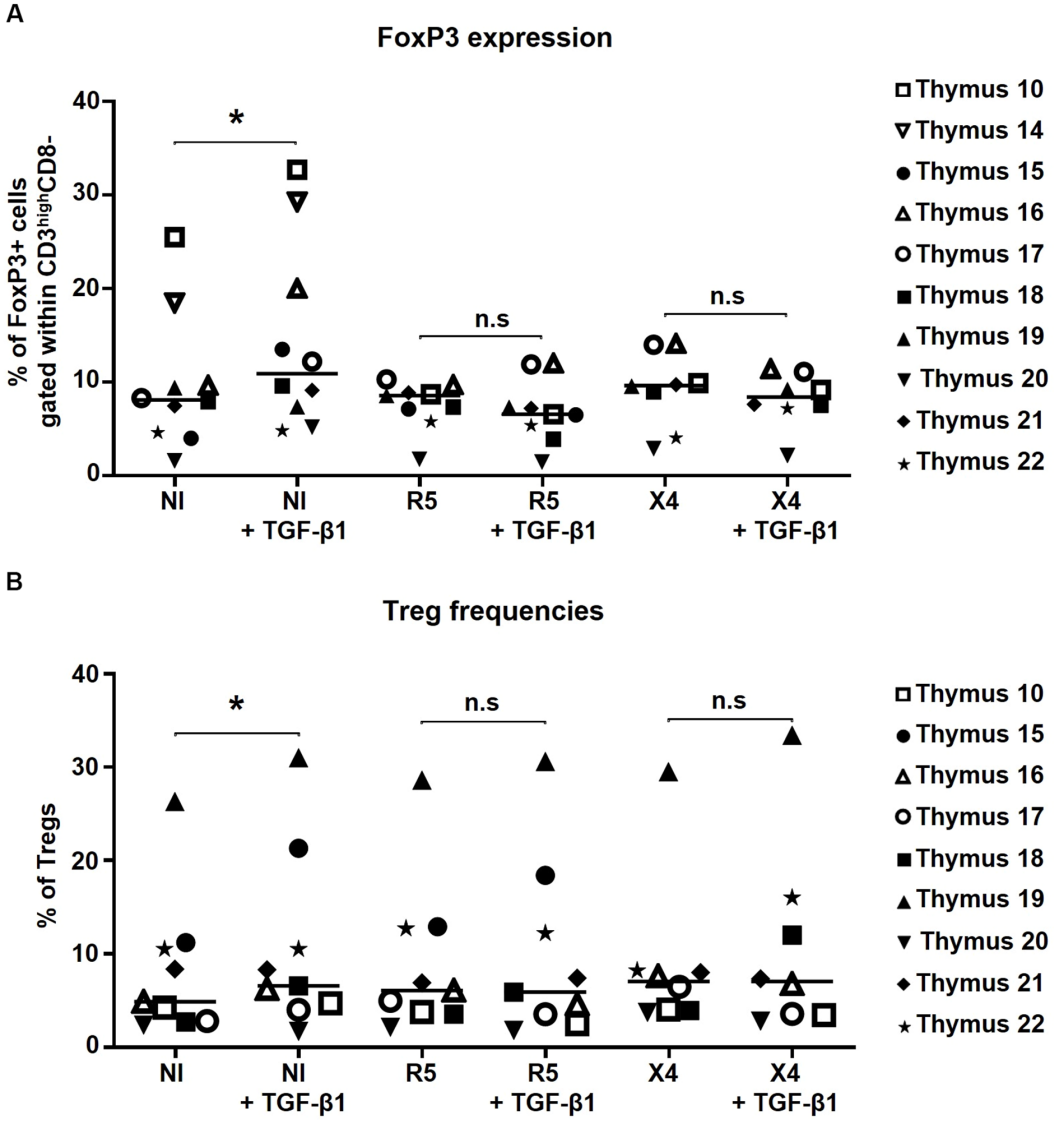
FoxP3 expression and thymic Treg frequencies are not altered by *in vitro* HIV infection alone or in combination with TGF-β1 treatment. **(A)** The frequency of FoxP3+ cells within CD3^high^CD4 + CD8- cells. **(B)** The frequency of thymic Tregs (CD4 + CD127-CD25 + FoxP3+) within CD3^high^ cells. Data from *n* = 10 **(A)** and *n* = 9 **(B)** thymi are presented. NI stands for non-infected controls. * *p* ≤ 0.05. n.s, non significant. *Nota bene*: While gating for CD4+ cells, CD4 + CD8+ thymocytes were excluded from the analysis.

To evaluate the effect of *in vitro* HIV infection on Treg generation, 1 × 10^6^ thymocytes were infected with R5- and X4-tropic strains in presence or absence of TGF-β1. Herein, neither FoxP3 expression by CD3^high^CD4+ T-cells (Figure 4A) nor the tTreg frequency (Figure 4B) changed upon *in vitro* HIV infection alone or in combination with TGF-β1 treatment, suggesting that thymocyte infection with HIV does not affect FoxP3 expression by CD4 T-cells or tTreg differentiation.

### *In vitro* HIV infection does not alter endogenous TGF-β production in thymocytes

**Figure 5:**
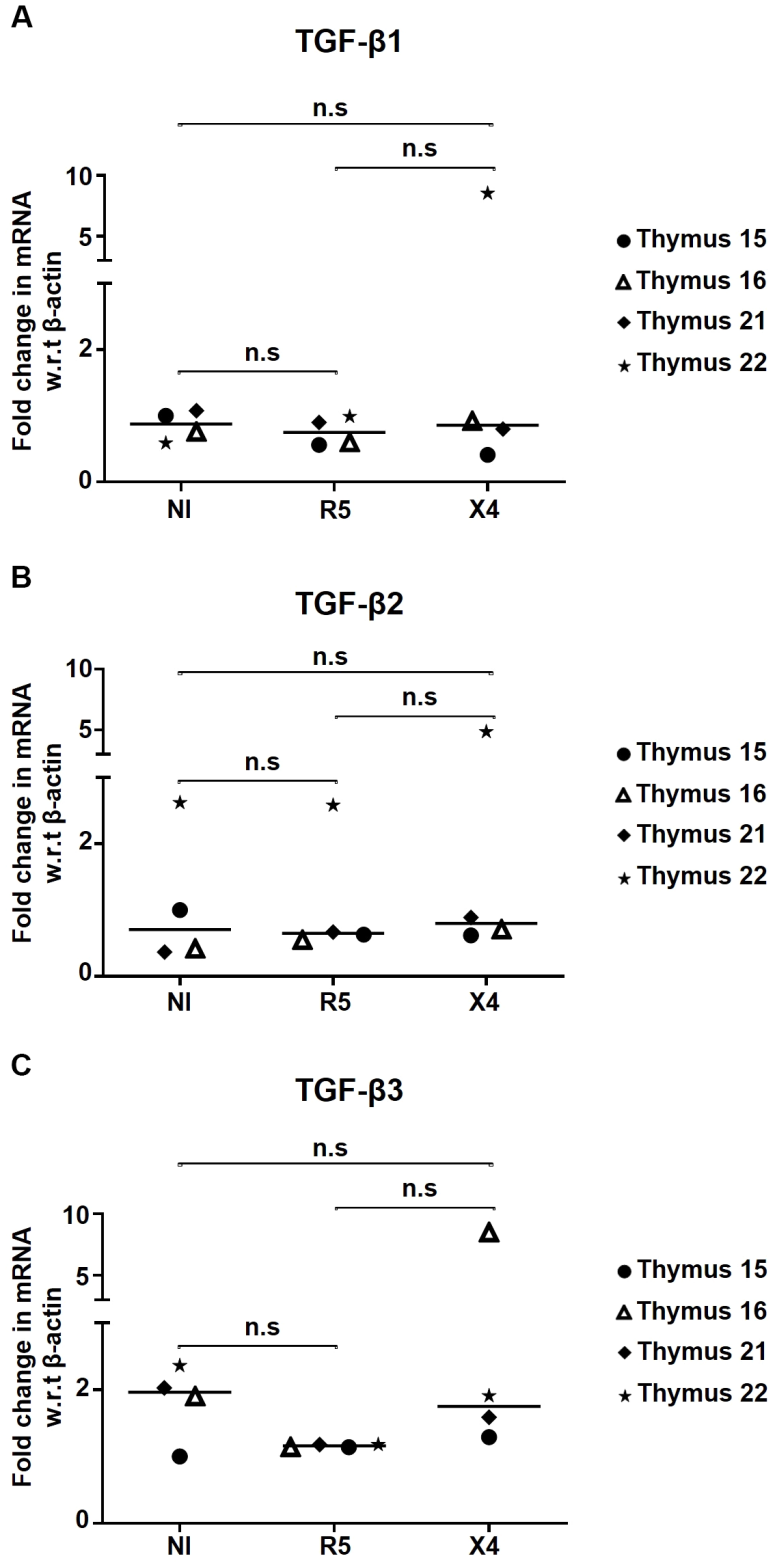
HIV infection has no influence on TGF-β production by thymocytes. Fold change in mRNA expression of **(A)** TGF-β1, **(B)** TGF-β2, and **(C)** TGF-β3 in thymocytes infected with R5- and X4-HIV-1 strains, compared to the non-infected control (NI) determined by qPCR (*n* = 4). No significant statistical difference has been observed within study subgroups. n.s, non significant.

Since Tregs are known producers of TGF-β during HIV infection, we wanted to evaluate whether *in vitro* HIV infection of thymocytes alters their endogenous TGF-β production. TGF-β1/2/3 mRNA expression relative to β-Actin was quantified in non-infected and HIV-infected thymocytes. Herein, no changes in mRNA expression of TGF-β1/2/3 were observed in HIV-infected samples compared to non-infected controls, suggesting that TGF-β production remains stable during *in vitro* HIV infection (Figures 5A–C).

## Discussion

Tregs can differentiate either as a T-cell lineage within the thymus (tTregs) or be derived from naïve CD4+ T-cells in blood and secondary lymphoid tissues during inflammation (Chen et al., 2003; Curotto de Lafaille et al., 2004; Curotto de Lafaille and Lafaille, 2009; Sakaguchi et al., 2013). Although Treg frequencies and their immunosuppressive activities increase in individuals with chronic HIV infection, and TGF-β is known to cause fibrosis of lymphoid tissues and disease exacerbation in such individuals, the direct effects of HIV alone or combined with TGF-β in the induction of thymic tTregs remains unknown. In this study, *in vitro* HIV infection of human thymocytes was performed to assess its impact on FoxP3 expression and differentiation of tTregs along with the influence of TGF-β.

We first assessed if tTregs are susceptible to HIV infection and whether infection of thymocytes could result in increased Treg frequencies. Our results showed that although the majority of thymocytes are CXCR4+, tTregs express high levels of CCR5 and CXCR4, suggesting a potentially higher susceptibility of tTregs to HIV infection. Notably, in contrast to our observation on tTregs from thymic tissue, expression levels of CCR5 and CXCR4 in circulating Tregs were previously shown to be comparable to those of effector CD4 T-cells (Moreno-Fernandez et al., 2009). Previous reports also indicate that thymocytes are more susceptible to *in vitro* X4 infection compared to infection with R5-tropic HIV strains (Nunes-Cabaço et al., 2015), which correlates with higher expression of HIV co-receptor CXCR4 compared to CCR5 in human thymocytes, while this was not significant in our co-culture model of HIV infection. We thus assessed the permissiveness of Tregs versus non-Treg thymocytes to HIV infection using a co-culture model of human thymocytes and the OP9-DL1 cell line (Ramsdell et al., 2006). One of the main reasons for using OP9-DL1 co-culture, instead of the *in vitro* stimulation of thymocytes via anti-CD3 or cytokines such as IL-2, is that such stimulations either increase FoxP3 and/or CD25 expression on CD4 T cells (Sereti et al., 2000; Wang et al., 2007). Furthermore, our team previously reported this system as an optimal model to study the differentiation of human thymocytes *in vitro*, as well as their exogenous stimulation or HIV infection (Young and Angel, 2011; Young, 2012). Our *in vitro* data indicate that tTregs are refractory to HIV infection, which could be related to the fact that the transcription factor FoxP3 may block HIV viral transcription by restraining the expression of NFAT (Selliah et al., 2008). In agreement with this, it was demonstrated that Tregs and effector T-cells isolated from peripheral blood of HIV-infected individuals without treatment, have comparable levels of HIV p24 *ex vivo*; although *in vitro* HIV infection of Tregs is lower compared to effector T cells (Moreno-Fernandez et al., 2009).

TGF-β1 regulates FoxP3 expression in the thymus and lymphoid tissues during inflammation (Chen et al., 2003; Li and Flavell, 2008; Chevalier and Weiss, 2013; Jenabian et al., 2013; Chen and Konkel, 2015; Jurberg et al., 2015). Overexpression of TGF-β protein and mRNA by peripheral blood mononuclear cells (PBMCs) from HIV-infected donors has been previously demonstrated (Hu et al., 1996; Wiercinska-Drapalo et al., 2004; Theron et al., 2017). Indeed, HIV-gp160 and HIV-Tat induced TGF-β expression and increased TGF-β levels in the plasma have been correlated with disease progression (Kekow et al., 1990; Hu et al., 1996; Reinhold et al., 1999; Wiercinska-Drapalo et al., 2004). A major negative effect of TGF-β (one of the main cytokines produced by Tregs) in HIV-infected individuals is its role in lymphoid tissue fibrosis as it enhances the function of fibroblast cells, which results in excessive collagen deposition and lymphoid tissue dysfunction (Nilsson et al., 2006; Zeng et al., 2011; Theron et al., 2017). Based on these facts, we postulated that TGF-β1 might contribute to the induction and maintenance of thymic Tregs during HIV infection. Co-culture of human thymocytes with OP9-DL1 cells in presence of recombinant human TGF-β1 led to increased frequencies of CD127 (IL-7 receptor)-expressing CD4 T-cells, which confirmed that TGF-β1 promotes CD127 expression (Ouyang et al., 2013). Importantly, compared to untreated controls, FoxP3 expression and Treg frequencies were higher in all TGF-β1-treated specimens, whereas the presence of TGF-β1 inhibitor PFD blocked this effect. Our results indicate that HIV infection, by itself, does not increase FoxP3 expression, nor does the combination of HIV infection and TGF-β treatment. Indeed, Treg differentiation during HIV infection depends on a combination of multiple factors including, among others, TCR stimulation, immune activation/inflammation, IL-2 supply, and TGF-β1 activity. In our *in vitro* experiments only TGF-β1 was added without TCR activation (anti-CD3/CD28) which might have affected our observations. Moreover, the mRNA levels of TGF-β in *in vitro* HIV-infected thymocytes remained unchanged. Of note, we observed relatively low infection rates in thymocytes, which could be due to the fact that laboratory-constructed viral strains rather than clinical isolates were used, which may explain the differences when compared to previously reported data (Young and Angel, 2011; Young, 2012). Our results indicate that TGF-β does not alter HIV infection rates *in vitro.* It is to be noted that *in vitro* infection only mimics an *in vivo* infection setting, and does not serve as an exact replica of an *in vivo* infection. We do not observe TGF-β production during *in vitro* infection possibly for the reason that Tregs remain unaffected by infection or probably because tTregs present in the thymus are not yet functionally active. Finally, we acknowledge that the relatively small sample size in our study could impact the outcome of the results. However, due to the rareness of these specimens, it was not possible to increase the sample size for some experiments.

Altogether, our results indicate that direct *in vitro* HIV infection of human thymocytes in absence of TCR activation does not increase FoxP3 expression and tTreg differentiation, nor does the combination of HIV infection and TGF-β treatment. Although our initial hypothesis about the impact of TGF-β on thymic tTreg differentiation during HIV infection was not confirmed, our results suggest that differentiation of thymic tTregs within the thymus is distinct from peripheral blood and secondary lymphoid tissues and additional inflammatory mechanisms may be involved in the differentiation and thymic output of tTregs during HIV infection.

## Data availability statement

The original contributions presented in the study are included in the article/Supplementary material, further inquiries can be directed to the corresponding author.

## Ethics statement

The studies involving human participants were reviewed and approved by Université du Québec à Montréal (UQAM, #216_e_453). Written informed consent to participate in this study was provided by the participants’ legal guardian/next of kin.

## Author contributions

M-AJ designed the study. SS, AY, and OF performed the experiments and generated data. SB and JA provided access to specimens. SS, TS, AY, and OF analysed, discussed, and interpreted results throughout the study. SS, TS, and M-AJ wrote the paper. SS, TS, AY, OF, SB, JA, and M-AJ contributed to the refinement of the study and reviewed study outcomes. All authors contributed to the article and approved the submitted version.

## Funding

This study was funded by the Canadian Institutes of Health Research (CIHR, grant MOP 142294) and in part by the AIDS and Infectious Diseases Network of the Réseau SIDA et maladies infectieuses du Fonds de recherche du Québec-Santé (FRQ-S) to M-AJ. SS was supported by an MSc excellence scholarship from UQAM’s foundation. AY is the recipient of an FRQ-S doctorate fellowship. M-AJ holds the tier 2 CIHR Canada Research Chair in Immuno-Virology.

## Conflict of interest

The authors declare that the research was conducted in the absence of any commercial or financial relationships that could be construed as a potential conflict of interest.

## Publisher’s note

All claims expressed in this article are solely those of the authors and do not necessarily represent those of their affiliated organizations, or those of the publisher, the editors and the reviewers. Any product that may be evaluated in this article, or claim that may be made by its manufacturer, is not guaranteed or endorsed by the publisher.
