# Supplementary material for: Impact of in vitro HIV infection on human thymic regulatory T cell differentiation
Source: Front Microbiol. 2023 Jul 20;14:1217801. doi: 10.3389/fmicb.2023.1217801 (PMC10400333; doi:10.3389/fmicb.2023.1217801)
Supplement: Supplementary file 1 [file Data_Sheet_1.docx]

**Supplementary Figure 1**: **The effect of the timing of cell culture of thymocytes on cell viability and rate of *in vitro* HIV infection**. Thymocytes were infected with 50 ng p24/mL per 10^6^ cells of 110NB (R5) or NL4.3 (X4) HIV-1, in a co-culture with OP9-DL1 cell line for 2 and 4 days. (**A**) Frequency of alive cells following 2 and 4 days of culture. (**B**) The infection rate of in vitro HIV-infected thymocytes after 2 and 4 days of infection. NI stands for non-infected controls. Data from n=7 thymi are presented.
