# Supplementary figures and images for: Impact of in vitro HIV infection on human thymic regulatory T cell differentiation
Source: Front Microbiol. 2023 Jul 20;14:1217801. doi: 10.3389/fmicb.2023.1217801 (PMC10400333; doi:10.3389/fmicb.2023.1217801)

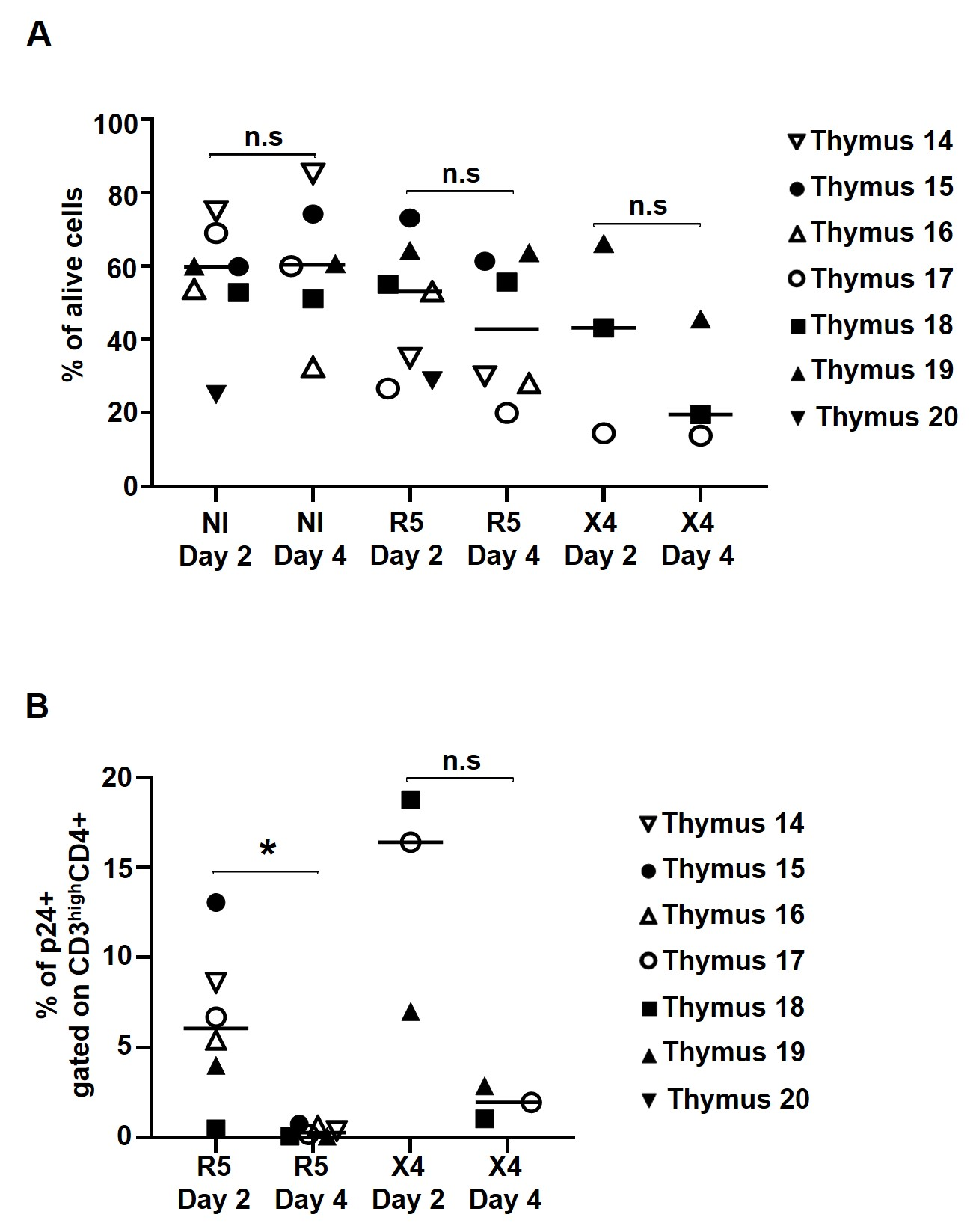

Supplement: Supplementary file 2 [file Image_1.tiff]
